# The mutual benefits of patient and public involvement in research: an example from a feasibility study (MoTaStim-Foot)

**DOI:** 10.1186/s40900-021-00330-w

**Published:** 2021-12-04

**Authors:** Alison M. Aries, Paul Bailey, Susan M. Hunter

**Affiliations:** School of Allied Health Professions, Faculty of Medicine and Health Sciences, Keele University, Staffordshire, ST5 5BG UK

**Keywords:** Patient and public involvement (PPI), Advisors, Value, Respect, ‘Public participation’, ‘Patient involvement’

## Abstract

**Background:**

Patient and public involvement (PPI) in research has increased steadily over the last two decades and is now both expected and appropriately resourced by many funding bodies, including the National Institute for Health Research (NIHR). However, PPI in research occurs in many different capacities and numerous frameworks exist for reporting or appraising patient involvement activities. The aim of this article is to describe processes involving PPI contributions to an NIHR-funded mixed-methods feasibility study (MoTaStim-Foot). Details of PPI advisors’ input, from initial identification and prioritisation of research ideas, to research delivery and dissemination, are discussed.

**Methods:**

Extensive PPI for MoTaStim-Foot is reported, with consideration of Research Design Service (RDS) advice for PPI for research, involving identifying and prioritising: design; grant proposal development; undertaking/managing research; analysing and interpreting; dissemination; implementation; monitoring and evaluation. Two PPI workshops were undertaken; success in meeting UK standards for public involvement was audited against specific success criteria by two researchers, with discussion and consideration regarding how well our PPI achieved inclusive opportunities, working together, support and learning, governance, communications and impact. How PPI can be improved for future trials was also considered. Although the advantages of PPI for researchers were considered, the benefits for PPI advisors were also analysed.

**Results:**

UK standards for public involvement were achieved, along with seven relevant research processes suggested by the RDS. PPI advisor contributions: informed study design; contributed to successful funding; enhanced trial delivery by informing participant information sheets and daily diaries; added value through undertaking note-taker roles in focus groups and helping to analyse focus group transcripts; and assisted in dissemination. However, benefits were mutual with PPI advisors reporting feeling valued and respected, a sense of pride with renewed confidence and purpose in life.

**Conclusions:**

Importance and value of PPI, to researchers and patient advisors, have been highlighted, reinforcing the benefits of working in partnership with PPI advisors.

*Trial registration* ISRCTN 13676183; Central Portfolio Management System ID 30449. Registered 02/01/2015, https://www.isrctn.com/ISRCTN13676183.

**Supplementary Information:**

The online version contains supplementary material available at 10.1186/s40900-021-00330-w.

## Plain English summary

This article reports and analyses our public involvement in the research processes for a study funded by the National Institute for Health Research, the MoTaStim-Foot feasibility study. MoTaStim-Foot explored whether it was feasible to deliver treatments to help people feel their foot better after stroke. The results of this study have been published elsewhere (1). Patient and Public Involvement (PPI) advisors played an important role. They worked voluntarily, actively advising the research team; the term ‘PPI advisor’ is used within this article, following consultation with our PPI advisors. They were involved during idea generation and discussions about whether the study was important to pursue. In addition, they advised the research team during the study setup helping to write the plain English summary, the participant information sheets and advised on other research activities. Following relevant training, PPI advisors were involved in delivering the research including note-taking during focus groups. One PPI advisor also helped analyse the focus group transcripts and attended the UK Stroke Forum to present the results of the study. Reflecting upon the impact of the PPI advisors’ contributions to the MoTaStim-Foot study has evidenced that not only was there great benefit to the research team and process, but our PPI advisors gained satisfaction from being involved in the research, planning, design and delivery. It made them all feel valued and respected and helped one of them find a renewed purpose in life. It is important to understand there can be mutual benefit from PPI advisors’ contributions to research.

**Supplementary Information:**

The online version contains supplementary material available at 10.1186/s40900-021-00330-w.

## Background

First described as an idea in 2007 by the Department of Health [2], ‘personal and public involvement’ has evolved over the years. Definitions for patient and public involvement (PPI), as it is now more commonly known, have been suggested, with Rise et al. [3] highlighting the importance of mutual respect and discussions to reach collective decision making. INVOLVE, a national advisory group commenced in 1996, funded by the National Institute for Health Research (NIHR), supported active public involvement in the National Health Service (NHS), public health and social research [4], but was taken over by the NIHR Centre for Engagement and Dissemination in April 2020**.** The NIHR define public involvement in research as "research being carried out ‘with’ or ‘by’ members of the public rather than ‘to’, or ‘about’ or ‘for’ them" (p 6) [5]. The inclusion of PPI advisors in research has been steadily increasing over the last two decades and researchers have moved from feeling uncomfortable and apprehensive as a result of what was an unfamiliar way of working with PPI advisors [6] to PPI advisors’ contributions being commonplace [7]. Indeed, PPI is expected for some funding bodies, for example, the NIHR [8, 9]. Allowing appropriate allocation of costs for PPI when submitting grant applications has facilitated opportunities for PPI advisors [10]. Various ideas exist for public involvement activities, and activities are often poorly defined in the literature [11]; consequently, evaluating the impact of PPI for research is challenging [12], although some useful models have been suggested [13]. In fact, a recent systematic review found 65 published frameworks to appraise or report PPI within medical research and the authors proposed that a locally developed bespoke frame-working process for PPI should be co-designed to address this gap [14]. Indeed, the value of such collaborative workshops with PPI advisors has already been demonstrated at Keele University when developing a training programme with our PPI advisors [15].

This paper will inform how PPI advisor contributions were successfully integrated within an NIHR funded randomised, mixed-methods feasibility study (MoTaStim-Foot) hosted at Keele University; the mutual value both to the research team and the PPI advisors will be discussed. This study was funded by the NIHR as part of a personal fellowship and, therefore, there was not a PPI advisor as a co-applicant; however, PPI advisor contributions were integral throughout the study. It is hoped that this example of public involvement within the research process will contribute to existing good practice case studies [16–18], enabling other researchers and PPI advisors to learn and begin to understand true mutual value of PPI. The aim of the PPI advisor contributions within the trial was to enable valuable insights from the perspective of a stroke survivor or carer during the complete research cycle, from designing the study to the implementation of findings, evaluation and dissemination phases of the research journey for the MoTaStim-Foot feasibility study, adding value and making the study more relevant and credible. Our PPI advisors were carefully selected from a pool of people expressing an interest in informing our research, and were able to contribute effectively to the research process because they are experts in the area of stroke, gained from their lived experiences [19].

### Methods

#### Aim, design and setting of the study

The MoTaStim-Foot study was a pilot and feasibility study in which two groups received sensory stimulation to the foot after stroke, in combination with task-specific walking training, to improve balance and walking. The study setting was an in-patient stroke rehabilitation ward and a community-based Stroke Early Supported Discharge service.

**Fig. 1:**
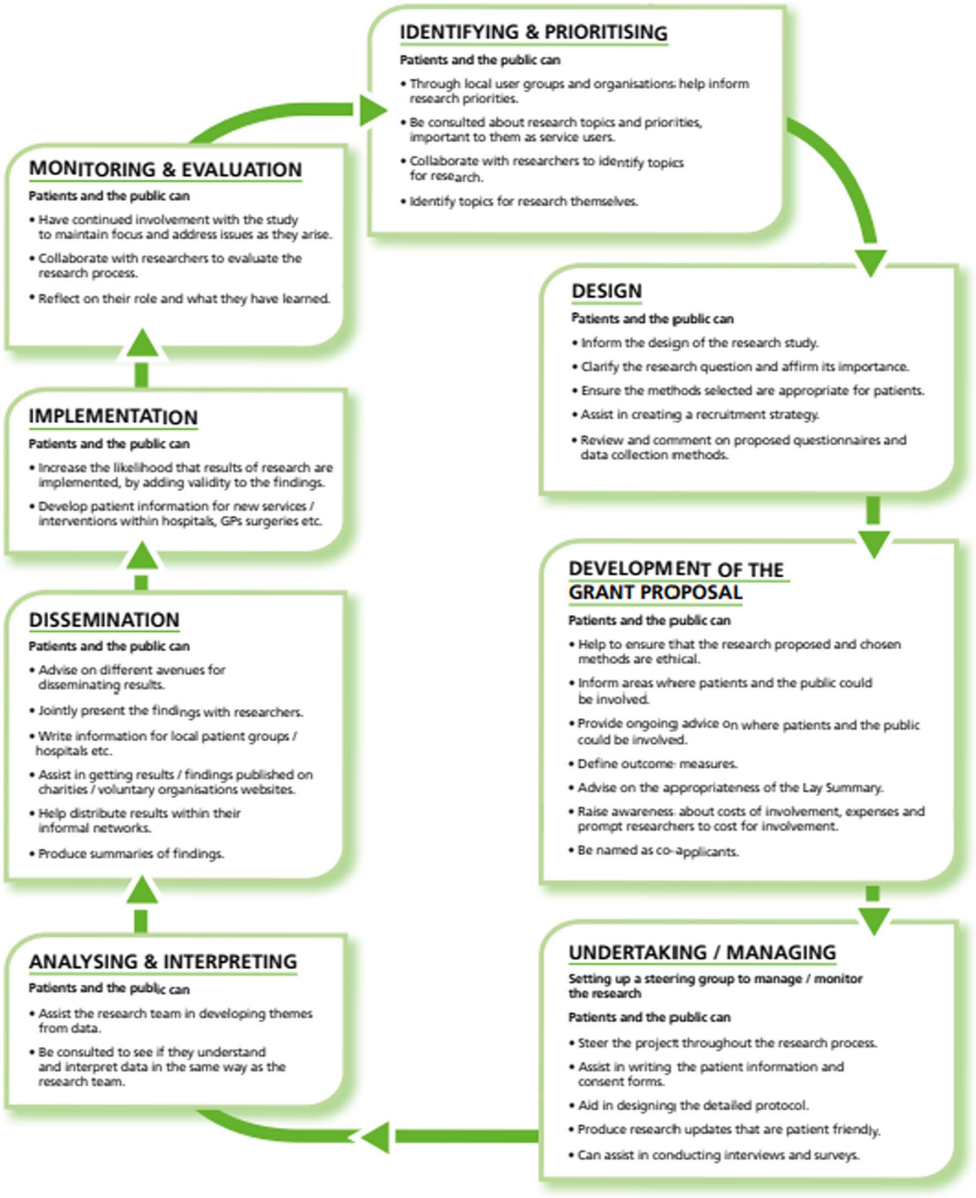
How to incorporate patient and public involvement in the research process ( reproduced with permission from the NIHR)

The MoTaStim-Foot study has been reported in detail elsewhere [1, 20]. The purpose of this follow-up paper is to describe the important role of PPI throughout the study and discuss the mutual benefits to the trial team and the PPI advisors. The different stages of PPI advisor contributions are detailed in the GRIPP 2 checklist [21] (Additional file 1) and will be discussed, highlighting the benefits and challenges from the perspective of both the PPI advisors and the research team. The advice offered in the RDS handbook for researchers (written by the NIHR, p14) [22] (Fig. 1) was also considered when implementing PPI within this study. Consideration was also given to how our PPI met the UK standards for public involvement: inclusive opportunities, working together, support and learning, governance, communications and impact [23]. We reflected on the questions posed for each section of the standards, and where we were able to answer "yes" to all but one question (e.g., four out of five (80%), or three out of four questions (75%)), we considered that we had met the standard. Details of how these aspects were addressed within the study and suggestions for how PPI can be improved in our work in the future were tabulated.

PPI advisors were involved in the planning and development of the MoTaStim-Foot study through four key stages: Stage 1 A series of interactive workshops; Stage 2 Application for funding; Stage 3 Pilot study; Stage 4 Dissemination of findings.

## Stage 1 A series of interactive workshops

### Workshop A (16/9/13)

PPI began several years before the MoTaStim-Foot study was commenced; the researchers’ initial thoughts and ideas were presented and discussed in a 90-min workshop with six stroke survivors (3–12 years post-stroke) and six carers at Market Drayton Stroke Club. The lead researcher (AMA) provided a presentation and demonstration of techniques considered appropriate to take forward as a research intervention. At the stroke club workshop, members were encouraged to give feedback on the proposed intervention and make suggestions about its suitability and appropriateness. This stage was important for “identifying and prioritizing” ([22], p. 14) the proposed research (Fig. 1). The group members were consulted about whether they thought the proposed research topic was appropriate or not and asked to give an indication as to whether they thought it should be a priority area for research or not. In view of the importance of the research for potentially helping people to walk again post-stroke all the attendees at the meeting felt it was an appropriate area for research and should be seen as a priority to be investigated. PPI advisors subsequently collaborated with us to refine the topic for research and consensus was reached during the discussions. Unfortunately, there was no additional funding available to offer remuneration to the stroke survivors and carers involved in this workshop for their time and insight, but they considered this to be an interesting item of their monthly meeting agenda and were happy to have given their time.

### Workshop B (24/4/14)

**Fig. 2:**
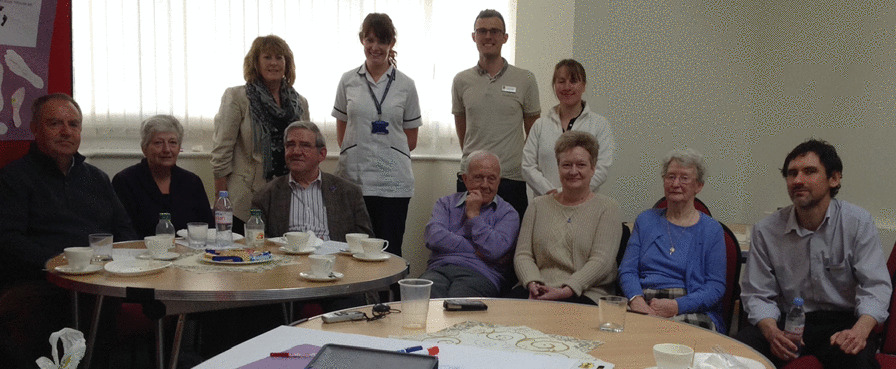
Photograph of the people attending the PPI workshop on the 24/4/14

A successful application for funding via an NIHR Research Design Service (RDS) PPI bursary supported further PPI advisor contributions to the development of a research project proposal and enabled appropriate remuneration for the PPI advisors’ time and valuable contributions. Although financial recompense was possible, all the PPI advisors chose to have a gift voucher instead as a way of saying "thank you"; travelling expenses were offered to all participants involved. This second workshop was arranged to inform the “design” of the proposed research (22, p. 14) (Fig. 1); this was attended by six PPI advisors, three experienced clinicians, and two academic members of the team (Fig. 2). The bursary funding was used to cover the PPI advisors’ travel costs to and from the community-based venue (church hall) and provided a buffet lunch and refreshments for the PPI advisors. Additional funding was found to enable lunch to be provided for the clinicians to say "thank you" for their time and wisdom and also for the research team to encourage team building with the PPI advisors and clinicians. PPI advisors included three stroke survivors (ME, MJ, NP) and three carers (AE, DT, AC). The project plans were presented by AMA and discussed by the whole group in greater detail. Clinicians and PPI advisors then attended separate, concurrent focus group discussions each lasting for one hour. During the focus groups, the participants were asked to consider the proposed outcome measures and interventions, with attention to potential burden and standardisation of outcome measures, frequency of treatments delivered, and type of intervention to be included in the feasibility study. The PPI advisors’ group was facilitated by AMA and the clinicians’ workshop was facilitated by SMH. PPI advisors and clinicians were then brought together to discuss key issues in more detail, ensuring the opinions of stroke survivors were thoroughly understood and considered when initialising the design of the study. An independent researcher experienced in PPI engagement (local NIHR RDS PPI advisor) was also invited to attend the discussion group with the PPI advisors and the joint collaborative discussion; he made notes on all the discussions to assist with ensuring that PPI advisors’ views were documented accurately and taken forward to inform the proposed study.

## Stage 2 Application for funding

Two PPI advisors (MJ and AE) expressed an explicit interest and were invited to contribute to the “development of the grant proposal” (22, p. 14) (Fig. 1) for an NIHR funding application for the MoTaStim-Foot study. They specifically reviewed the plain English summary, the protocol and participant information sheets for the study; the advisors made a few minor suggestions to develop appropriate wording to ensure that the documents were clear and the language was not jargonistic, and to offer their opinions on the appropriateness of the protocol for stroke survivors. All changes to wording were agreed between the lead researcher (AMA) and the PPI advisors through discussion; there were no disagreements.

## Stage 3 MoTaStim-Foot pilot study

Following success of the NIHR funding application, the MoTaStim-Foot study was set up between March 2015 and March 2016, and the first participant was recruited in May 2016; recruitment continued until November 2017. Three PPI advisors (AE, JJ and PB) were keen to have further involvement with the study. Their preferences for involvement were discussed and agreed with the lead researcher (AMA), and they subsequently were welcomed as key members of the research team, attending the trial management group (AE, PB), assisting with data collection (JJ, PB), and analysing data (PB). Appropriate means of communication were adopted for each PPI advisor according to their individual preference. For two of them (AE and PB) this involved email; however, one other (JJ) was not a computer user and, therefore, other more effective communication methods were adopted, including telephone calls and postal services, to ensure inclusivity as appropriate. 

**Fig. 3:**
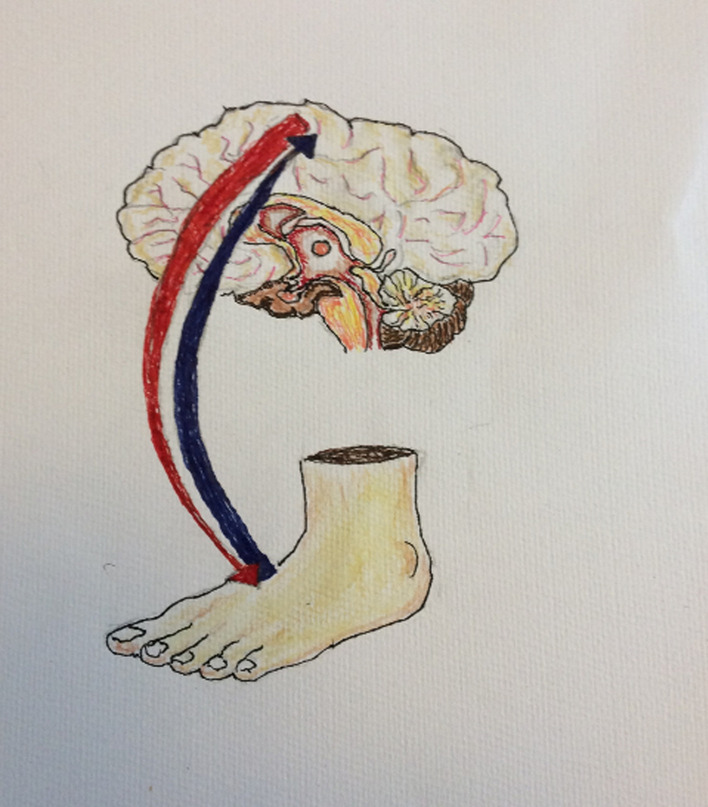
Sensory feedback influencing motor control

**Fig. 4:**
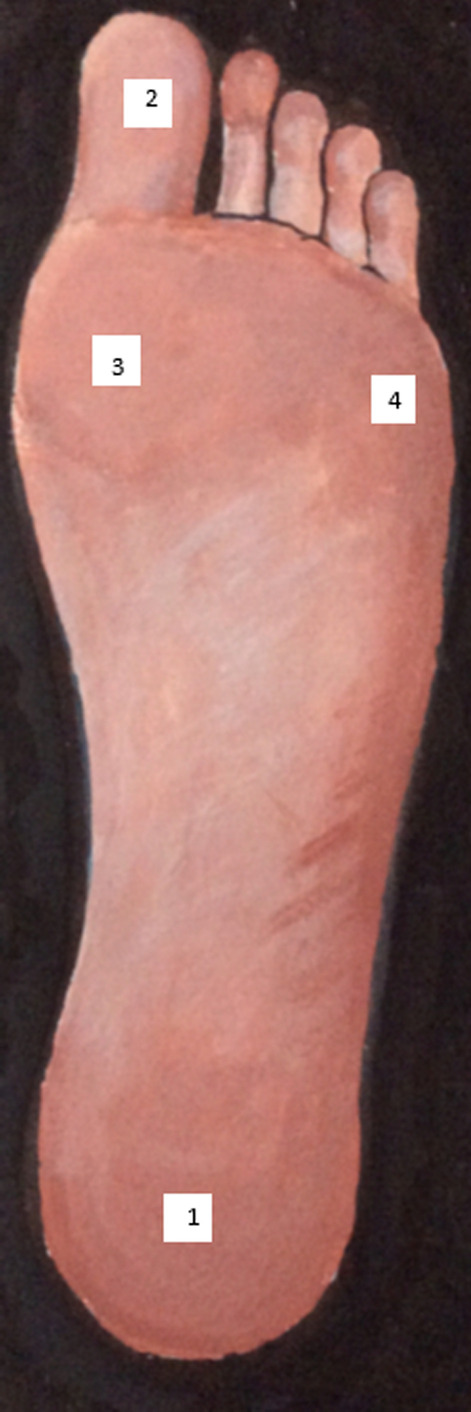
Points tested with Semmes Weinstein monofilaments

**Fig. 5:**
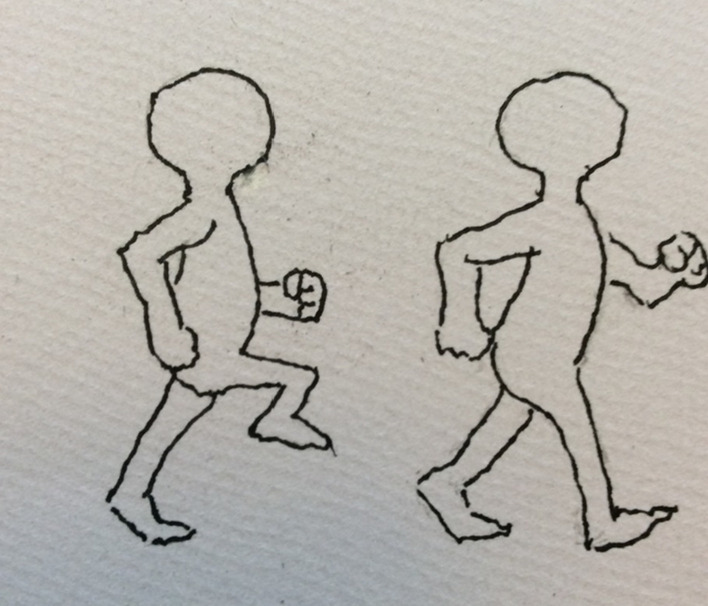
Task specific walking training

PB used to teach art prior to his stroke. Despite losing the ability to draw with his dominant hand, he successfully developed several images to assist with the research documentation. For example, an image depicting how sensory feedback influences motor control (Fig. 3), a picture showing the points on the soles of the foot that were tested with the Semmes Weinstein Monofilaments (Fig. 4) and a picture representing the task-specific walking training intervention (Fig. 5).

AE and PB were appointed as members of the Trial Management Group (TMG), attending regular meetings to oversee the study, fulfilling aspirations to have PPI advisors involved with “undertaking/managing” and “monitoring & evaluation” (22, p. 14) of the research (Fig. 1). PPI was a standing agenda item for the TMG. To assist with PB’s ability to undertake the role, he attended a face-to-face Good Clinical Practice (GCP) training session, whereas it was not appropriate for AE to attend GCP training due to caring commitments for his wife (ME), and he was, therefore, supported informally on an individual basis by the lead researcher (AMA).

Two PPI advisors (JJ and PB) were also trained to undertake the role of note-taker for focus groups with participants (stroke survivors) in the MoTaStim-Foot study. They attended between one (JJ) and three (PB) focus group discussions as a member of the research team, collecting supplementary data in the form of hand-written notes based on observation of behaviours, and summarizing key issues that arose. Debriefing sessions were held after each focus group, involving the PPI advisors, senior members of the research team involved in the focus groups, and the group facilitator (AMA). This enabled further discussion relating to the main topic areas and preliminary themes [24], and identification of missing topic areas [25] that should be considered for exploration in future focus groups. Another purpose of the debriefing meetings was to offer psychological support to the PPI advisors in case of unpredictable emotional response [26] that could have potentially been triggered by comments made within the focus group that may have elicited memories of their own stroke experience and journey.

PB also contributed in his capacity of PPI advisor as a member of the research team analysing the focus group data once it had been transcribed. The transcriptions and initial analyses were shared with PB, which he read and subsequently offered additional insights to the analysis through discussions with the wider research team, contributing to development and agreement of the final themes. As a novice qualitative researcher, it was deemed to be more appropriate to invite PB to be part of the analysis at the second stage. Initial analysis was undertaken by three experienced qualitative researchers. This important PPI advisor contribution fitted the remit of “analysing & interpreting” as recommended in the RDS PPI handbook for researchers (22, p. 14) (Fig. 1).

## Stage 4 Dissemination of findings from the MoTaStim-Foot study

The findings from the MoTaStim-Foot study were presented during an interactive poster tour by AMA at the United Kingdom Stroke Forum in December 2018. PB was a co-presenter with AMA, and he played an active role in explaining the study and the findings to interested conference delegates.

## Results

The impact both for the study and the PPI advisors is presented for each stage of the MoTaStim-Foot study. Table 1 summarises how the different aspects of the UK standards for PPI (inclusive opportunities, working together, support and learning, governance, communications and impact) [23] were met and could be improved in relation to the MoTaStim-Foot study. Success in meeting the standards was achieved, based on being able to answer "yes" to the proposed success criteria (questions), (able to answer at least four out of five (80%), or three out of four (75%) questions posed in each section).

**Table 1:** How our PPI met the six UK standards, and how it can be improved

UK Standard	PPI meeting this standard	How PPI could have been improved
Inclusive opportunities	Workshops A and B were held at an early stage in the research process Male and female advisors, stroke survivors and carers Workshops held in non-threatening, familiar environments e.g., local church hall Remuneration for time and travel offered	Increasing diversity within our PPI advisors would be beneficial. All our PPI advisors were white, British, middle to older aged stroke survivors or carers
Working together	Practical arrangements to facilitate shared working were employed Opinions of PPI advisors were valued and respected and actioned where appropriate	The purpose of PPI within the study could have been openly discussed with PPI advisors with collaborative decision making and documented appropriately
Support and learning	One PPI advisor accessed GCP training Two PPI advisors were trained to undertake the role of notetaker in the focus groups and one advisor trained to assist with analysis of the focus group	PPI advisors could have been invited to a meeting to discuss their learning needs
Governance	There was PPI advisor representation at all of the trial management meetings, with PPI being a standing agenda item Advisors’ opinions were listened to and valued Remuneration for PPI advisors’ attendance at meetings was built into the funding	Training to ensure PPI advisors were able to be assertive in meetings may have been valuable and should be considered in the future
Communications	Appropriate communication methods were used according to advisors’ preference e.g., offering to post out hard copies of documents PPI and achievements are being shared	Initial meetings with PPI advisors could establish preferred communication methods, so all are aware
Impact	This article has been written in collaboration with one of our PPI advisors The importance of PPI advisors’ contributions was highlighted when one of our PPI advisors presented with AMA (the lead researcher) at the UK Stroke Forum The researchers have become even stronger advocates of PPI than previously, with greater insight of the benefits	An informal meeting with all the PPI advisors when the study was completed would have been valuable to help understand their opinions about their involvement in the research cycle for this study and to feed into further improving the PPI advisor’s contributions for future studies

## Stage 1

### Workshop A

During workshop A, stroke club members agreed that being able to feel their feet accurately was important for function e.g., balance, standing and walking. Verbal consensus was gained through informal discussions, ensuring everyone’s opinion was heard, and the group advocated that the research should be taken forward. One stroke survivor (MJ) felt a lack of sensory feedback to the foot increased the chance of tripping stating:

"*On my part I still remember the difficulties I experienced with my affected foot and to a degree still experience them today, nine years post stroke. Loss of sensitivity and on occasion hyper-sensitivity made/makes walking a problem. I am absolutely certain it is an appropriate area for future research".*

As a result of the consensus from the group, the research team were reassured that the proposed project was relevant to stroke survivors and felt it would have credibility with service users and carers. The involvement of PPI advisors at this stage was valuable to the research team; however, it was also of clear benefit to the stroke club members, who felt that their opinion and experience was respected (evidenced by post-event feedback by email from the group chairperson) and their input to the project ideas at this stage would be used to develop a better and more credible research project. MJ became an active member of the research team from this point on.

### Workshop B

At workshop B, new and useful suggestions were made for consideration during the protocol development stage. In particular, these included: fatigue management; types of shoe to be worn; and potential burden of the outcome measures, which contributed to finalising the choice of measures for the study.

The research team had proposed that stroke participants in the MoTaStim-Foot study should maintain a daily diary of their experiences of the study and any subjective changes noted in their foot or balance/walking; feedback from one PPI advisor suggested that the diary should include clear prompts to make it easier for stroke survivors to complete.

"*To help people fill it in, keep it simple*".

This important advice was taken on board when developing the study, and the daily diaries were constructed with clear verbal and visual prompts to facilitate completion. The understanding from PPI advisors that completing a daily diary without prompts was something that the research team had not considered specifically, and this insight from PPI advisors was of great benefit to the study participants (stroke survivors) who were subsequently asked to record their subjective experiences on a daily basis throughout the study.

## Stage 2 Application for funding

PPI advisor contributions from MJ and AE were essential to inform the funding application for the study. Guidance relating to wording of the plain English summary was particularly useful, ensuring appropriate terminology was used. MJ was extremely passionate about this research and instrumental, with assistance from AE, in helping to develop the MoTaStim-Foot protocol and essential documentation, such as the participant information sheets for the study, once funding had been secured. Such was the success of this aspect that comments from the Research Ethics Committee complimented the role of PPI advisors within the MoTaStim-Foot study. Sadly, MJ passed away before the study was completed; however, his wife (JJ) continued as an integral part member of the research team, along with AE and PB, who is co-author on this paper. The valuable suggestions from the PPI advisors strengthened the application helping to secure funding. Equally, fulfilling the role of PPI advisor for the application stage was beneficial for the advisors, with the value and meaning of this role clearly expressed in the vignettes. MJ reported to his wife that he felt “*a sense of pride and satisfaction*” and that he had “*a purpose*” and felt it was “*rewarding… to be part of such a great team*”.

## Stage 3 MoTaStim-Foot pilot study

PPI in the MoTaStim-Foot study was an important component and was fully integrated into the study management and overall research process. There were many ways in which the PPI advisors’ contributions added value to the research process. PB was an art tutor prior to his stroke and, despite the fact he had lost the use of his dominant right hand, he produced some exceptional images, using his left hand, that were helpful when presenting and explaining information about the study, including the importance of sensory information to drive motor control (Figs. 3, 4, 5).

He also drew images to help with presenting information relating to the study interventions, which were included in written reports of the study [20].

Specific training was provided for PPI advisors, including a half-day session to train two PPI advisors (JJ, PB) to be able to undertake the role of note-taker, and further informal training to enable PB to undertake the additional role contributing to the analysis of the focus groups and development of the final themes. Both PPI advisors appreciated the training they received to help them fulfil their roles within the research team. PPI advisor contributions were costed and funded within the NIHR application and this enabled appropriate remuneration to the PPI advisors, based upon INVOLVE guidelines [4], for both travel costs and their time. Although money was offered, all PPI advisors preferred the option of taking a gift voucher instead as a "thank you" for their time. The training included NIHR Good Clinical Practice (GCP) training, which was completed by one PPI advisor (PB), covering the “international ethical, scientific and practical standard to which all clinical research is conducted” [27]. When completing the GCP training, PB had the confidence to remind the facilitator to refrain from using technical jargon and abbreviations, to increase his ability to understand the information delivered within the course.

The notetaker role was successfully carried out by two PPI advisors (PB and JJ) for the four focus groups undertaken in the study. JJ initially felt “out of her depth” and "threatened" by being involved because she was  not sure that she had the skills: she had never had any academia within her life, and this role was new to her. JJ reported that she liked to learn from other PPI advisors (PB) and felt supported by the research team. The added insight from the PPI advisors was extremely valuable. PB and JJ were able to empathise with the participants (stroke survivors) and ‘looked out’ for those who were perhaps reluctant to communicate their ideas, helping to facilitate their input. As a carer and a stroke survivor, both PPI advisors complemented each other well, with both bringing experience from a different point of view and two ‘separate sets of eyes’. As a carer, JJ was looking more at the stroke survivors’ and carers’ needs, whereas PB (a stroke survivor) was looking more at the effects of stroke on the stroke survivors. Furthermore, their input to the summary section of the focus group discussions was invaluable; they could put themselves into the shoes of the stroke survivors, adding a different perspective, and ensuring any aspects which may have been overlooked by the researcher (AMA) were included. Including PPI advisor representation in the post-focus group debrief meetings also proved useful, enabling initial analysis of the focus groups to be commenced, with PPI advisor input.

However, it is clear from PB’s vignette that being able to successfully achieve the note-taker role in the focus groups was an important aspect in his own recovery post stroke. The sense of achievement he felt boosted his own confidence and he stated that “*it made me feel really good contributing to something so important*”, and it was "*good to be part of a team again".* This helped him realise that, whilst the stroke had affected him physically, it had “*not hampered his ability to get involved in research and to help other stroke survivors”*. PB found the whole experience to be "*uplifting*", with "*no negatives whatsoever*". This sentiment was repeated by JJ as well. Their peer relationship was mutually beneficial despite their different experiences of stroke and their backgrounds. PB benefitted from meeting someone who had previously been involved as a PPI advisor and found it useful to speak to someone who had life experience of living with someone who had had a stroke. JJ was, therefore, experienced from a different point of view. JJ also reported that she found it helpful to work together and stated that she "*liked to learn from"* PB too.

## Stage 4 Dissemination of the findings of the MoTaStim-Foot study

**Fig. 6:**
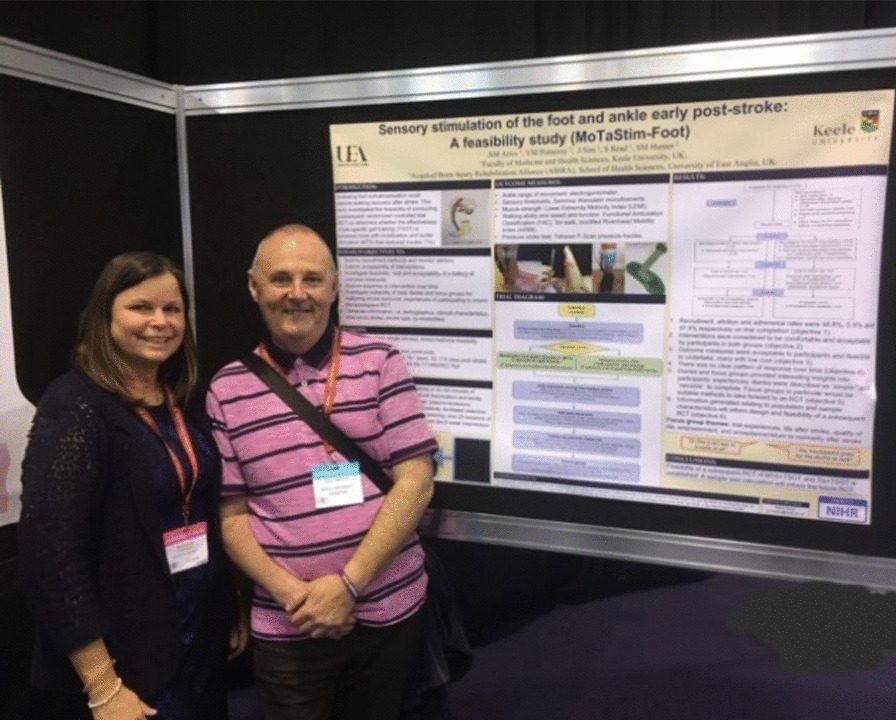
Presenting at the UK Stroke Forum

Having a stroke survivor (PB) involved when presenting the results of the study at the UK Stroke Forum added another dimension, a stroke survivor’s perspective, to dissemination (Fig. 6), and met the RDS recommendations for PPI [22] (Fig. 1). The conference delegates were keen to question him and find out more about his role within the study team.

**Fig. 7:**
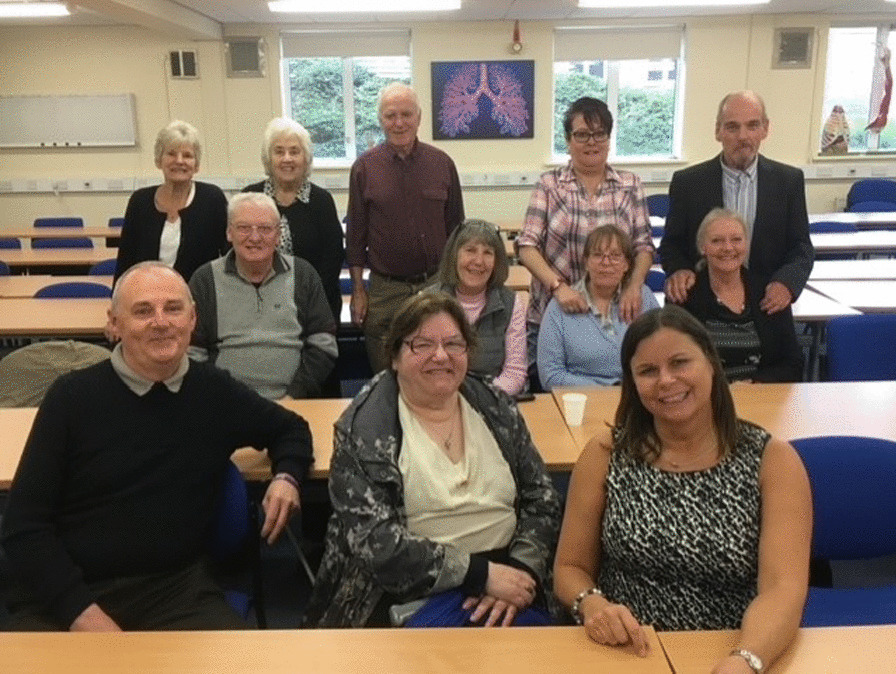
Participants present for the feedback session about MoTaStim-Foot

The results of the MoTaStim-Foot study were also reported back to the study participants; they were all invited to a presentation at the University, with lunch and refreshments provided. A total of six participants and three carers attended (Fig. 7). In their position of PPI advisors for the study JJ and PB attended this feedback session. Although there was no formal role for them at this presentation, they were integral members of the research team, and it was considered that their presence at the meeting would be useful in case any participants had questions they wanted to address to them specifically as PPI advisors; it also gave an opportunity for those attending to find out more, through informal discussions over lunch, about the role of being a PPI advisor within our research team. Several participants from the MoTaStim-Foot study expressed an interest in undertaking this role, as well as participating in further research studies in the future. It was important for the PPI advisors to be invited and be part of this meeting, recognising the accomplishment of the MoTaStim-Foot study and the role they played in making the study successful. It also gave us another opportunity to thank them for the role they played in the study, helping to ensure they felt valued and respected.

The ability to be an important ‘cog in a wheel’ again, and feeling appreciated, comes across clearly in the PPI advisors’ vignettes below:

Vignette 1 (PB):

*"Back in January 2017 I was asked by Alison if I would like to be a patient and public input (PPI) volunteer on a study that Keele University were doing called MoTaStim-Foot. I had been recommended by the physios from the [Haywood] hospital because of my attitude and determination to recover and to help in any research that might help myself or others. My background and job (a prison officer/tutor) meant communication was a strong point, I also taught art and although right-handed/right side affected, I managed to retrain my brain to produce paintings and drawings with my left hand. Some of these were produced to use in the research documentation. I was eager to help in any way I could and took any task given.*

*I was given the chance to increase my knowledge by doing GCP training at a hospital along with a group of hospital staff; it was at this stage that I felt out of my depth when I began to read the PowerPoint slides, but I didn’t suffer in silence and asked if they could stop using abbreviations that only mean something to people who work as medical professionals. They apologised and the whole day was very useful, learning things like how participants were recruited and how information was recorded and handled safely and securely.*

*My first task was a trial management group. I’m a people person that loves to meet and interact with others, so this study was a good thing to be involved with, as I feel it helped me put some things into perspective and share my experience with others to further their understanding of stroke and the effects on one’s life. Being a note taker was an interesting role, spotting people that weren’t engaging was a vital part whether that was because they were unable to do so because someone was too eager to share, or they were not comfortable due lack of confidence or communication difficulties due to their stroke. I found this very useful and became very good at it towards the end; it made me feel really good contributing to something so important. I found that doing the PPI role increased my need to help, so I became a volunteer with the Stroke Association and later became a stroke ambassador. I also got involved in a community-based music making group (STROKESTRA®-Stoke) which gave me a chance to take part and improve certain things that I set as targets, things that I felt were affecting my life due to stroke, and meet some extraordinary people who were living with similar things, learning how they cope and how their life has changed. I personally feel that my life has improved, and I have met some great people and learnt things about myself that have made my life easier and improved the life of others".*

Vignette 2 (MJ and JJ’s story, told by JJ)

MJ: *“Mel became a stroke survivor at the age of 55 years. He was told ‘"you may never walk again, you may never drive again and you may never work again"’. He did walk and drive again; however, he was unable to work again as an ophthalmic optician. Mel was passionate about getting stroke survivors back into useful lives. Being a stroke survivor himself, Mel knew the importance of research and trials and was so delighted to be asked to be a PPI volunteer for Alison. He always had a spring in his step when he went to Keele, enjoyed the company of the staff and patients, and felt a sense of pride and satisfaction, also a purpose using his knowledge. Mel knew the research trial would benefit his fellow stroke survivors, and had he lived, he would have seen their success. He would return home and tell me how rewarding it was to be part of such a great team, and he knew every effort was being made to make trials happen, I know he enjoyed ‘taking part’ at Keele. Sadly, Mel died in May 2016, never to know the outcome of Alison’s trial; however, he would have been so proud of Alison’s achievement”.*

*JJ: “T* *his is now where I came into Alison’s life, she asked me if I would like to be a PPI volunteer. I have no qualifications, only life skills, so felt very honoured to be asked, but I did feel ‘out of my depth’ as I’m not an academic, learning isn’t easy. The training given was useful and with each PPI session I became more comfortable and grew in confidence. I saw patients not able to stand or walk, given insoles to stimulate the foot and physiotherapists working on the foot, blossom into being able to walk and do things we take for granted not only physically but mentally. Observing what clinical trials do from my perspective was an eye-opener, and I only hope the department can continue in more research and clinical trials. I know from my personal experience there is ‘LIFE AFTER STROKE’, I would continue to be a PPI volunteer if asked”* *.*

The details regarding how the PPI advisors’ contributions to the MoTaStim-Foot study met the UK standards for patient involvement, and which aspects can be improved for the future are summarised in Table 1.

In summary, there were numerous positive outcomes resulting from the PPI advisors’ contributions to the MoTaStim-Foot study. PPI advisors helped to shape the research design and documentation, input into how the research was undertaken via their role in the trial management group, assist in data collection and dissemination of the results. There were no specific negative effects to having such an intensive level of PPI for the study; however, additional strategic planning and finance were necessary to ensure appropriate lines of communication were used and that PPI advisors were appropriately compensated for their time supporting the study.

## Discussion

The PPI advisors played an important role within the research team for the MoTaStim-Foot study. Their input was valuable right from the initial inception of the research ideas through to the dissemination of the work. Indeed, seven of the eight suggested ideas for incorporating PPI in the research process included in the RDS handbook for researchers (written by the NIHR, p. 14) [22] were fulfilled within this study process (Fig. 1). The idea not included was implementation, which would not have been appropriate; this was early-stage research, designed as a feasibility study.

It was particularly insightful to have a stroke survivor and carer as research team members when summarizing the focus group discussions and seeking verification from the participants. It brought a deeper level of understanding, by virtue of their ability to understand and empathise appropriately about how it feels to be a stroke survivor.

Respect for the PPI advisors’ opinions relating to the progression of the study was evident from everyone in the study team; for example during the trial management group meetings, where the opinions of PPI advisors were listened to and considered, and where suggested changes were agreed by the whole team following discussion, these were actioned [5, 9].

Researchers and PPI advisors all agreed that there were no specific challenges to report associated with the PPI advisors’ contributions to the MoTaStim-Foot study; however, it is acknowledged that there were occasions when PPI advisors’ advice could not be followed. This could be perceived by PPI advisors that their opinion was not valued. An example of this was that in workshop B when it was suggested that footwear was standardized when undertaking the outcome measures. It was not feasible or appropriate to provide standardized footwear for all the participants in the study; however, this suggestion was considered. As a compromise it was ensured that each participant wore the same footwear on all occasions when undertaking the baseline and outcome assessments.

It is important that PPI advisors are offered appropriate  remuneration for their time and travel expenses [5]. It was possible to ensure payments were offered to all our PPI advisors, because PPI advisors’ contributions were appropriately costed and funded for this study, based on the INVOLVE recommendations [9].

We acknowledge a limitation relating to the diversity within our PPI group of advisors. This may have meant that the opinions and needs of stroke survivors from other cultures and age groups were not adequately represented within our study.

Our approach to PPI in research fits well with the UK standards for public involvement [23], facilitating working together and implementation of appropriate support and learning, good communication, enabling PPI advisors to influence governance of the research and evaluating the impact of PPI. After working closely with the PPI advisors and conversing informally on regular occasions, it was evident that it was not only the research team and processes that benefitted from their input; PPI advisors also gained a great deal from feeling like they were contributing to the research process. PB benefitted from being a valued and active member of a team again, which not only gave him a reason to leave the house but restored his faith in his ability to be a useful member of society and, through his contributions, to benefit other stroke survivors. His experience was described as being "*uplifting*" and he was adamant that he was unable to identify any negative aspects associated with his input. The confidence that PB gained by being a PPI advisor even facilitated his next step on his road to recovery post-stroke, prompting him to volunteer for the Stroke Association and later to become a Stroke Ambassador. For JJ, who was initially daunted by being invited to contribute to research as a PPI advisor, her experiences were again beneficial; she has reflected on her ability to better understand research and stated that she wished she had been involved in that capacity earlier on in her role as carer.

Another aspect to consider is the researchers’ perspective and what was learnt or gained from working with PPI advisors. Some researchers have disclosed a better understanding of the experiences of the people living with the condition being researched [28]; however, as both the key researchers are physiotherapists with many years of experience working with stroke survivors it was perhaps not an expectation that "experiential knowledge" ([29] p. 7) would increase. One aspect that we were perhaps made more aware of was the importance of using plain language and avoiding acronyms when working with PPI advisors. It has been advised that the use of abbreviations is kept to a minimum and an explanation given when they are first used, short sentences are used, and technical terms avoided when developing documentation for health [30]. Our recommendation is that this approach is used when working with PPI advisors too.

Our whole research team and the wider governance group within Keele University believe it is important to build relationships over time and to encompass PPI advisors as significant, respected, and valued members of the research team; these factors have been acknowledged previously as being important when working with PPI advisor members [10].

In summary, from our experience of the MoTaStim-Foot study and learning about the mutual benefits of PPI, the following are key aspects of PPI in research that we will endeavour to continue to include in our own research:

- Early involvement of PPI advisors in discussions about the planned research
- PPI advisors’ advice when writing the plain English summary
- Appropriately funded remuneration for PPI advisors’ time and other expenses
- Full integration of at least one PPI advisor into the research team e.g., Trial Management Group member
- Involvement of at least one PPI advisor in interpreting research findings
- Provision of appropriate (and funded) training for PPI advisors to fulfil their roles
- Co-presentation and dissemination of research findings at high profile national or international conferences, with full funding provided to cover costs

## Conclusion

Reflecting upon the PPI within the MoTaStim-Foot study has given an opportunity to fully appreciate the importance and value of PPI advisors’ contributions to both the research team and the PPI advisors, further fulfilling the “monitoring & evaluation” aspect of incorporating PPI in the research process (22) p. 14). This report has highlighted that it is possible to work in partnership with PPI advisors, with them influencing the research process at all stages from initial ideas to delivery and dissemination of research findings.

**Fig. 8:**
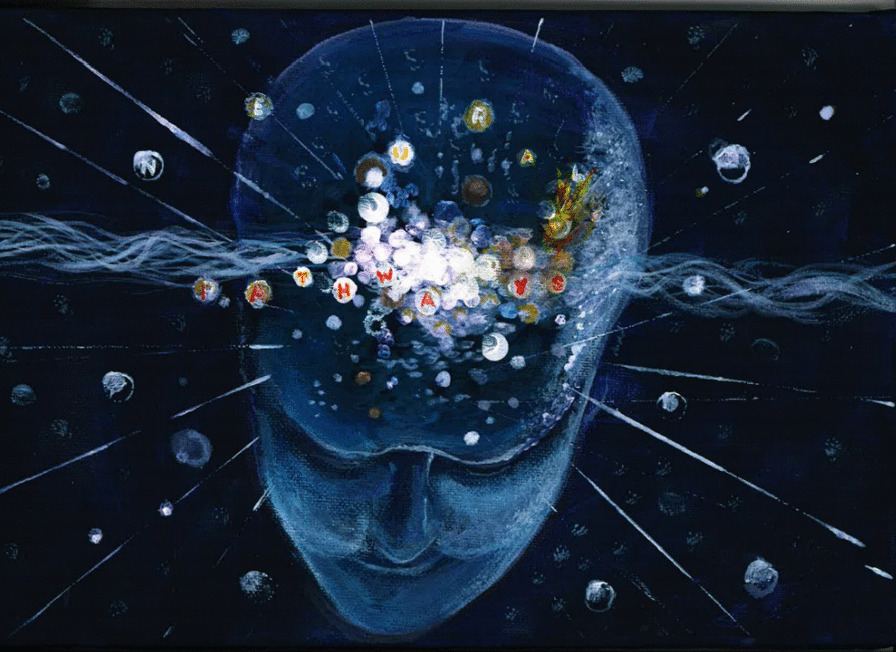
PB’s image of his own brain post-stroke

This exemplary PPI advisor input has not only improved the credibility of the research; it has also enhanced the PPI advisors’ perception of their own value and importance within the research process, giving added purpose to their life after stroke. PB appreciated being respected and valued within the research team and, as a gift to demonstrate his appreciation of his experience working as a PPI advisor for MoTaStim Foot study, he gave AMA an image he had drawn depicting his own brain and the effect of his stroke. The image portrays the reforming of neurological pathways (Fig. 8); this is exactly what the sensory stimulation delivered within the MoTaStim-Foot study aimed to achieve. The mutual benefit of PPI advisor input within our research is a shared mission that we plan to continue in the future, and an aim we hope others will also adopt.

## Supplementary Information

**Additional file 1.** The GRIPP 2 checklist.

## Data Availability

All data generated or analysed during this study are included in this published article. The PPI discussed was for the MoTaStim-Foot publication [1].

## Abbreviations

GCP: Good Clinical practice
NHS: National Health Service
NIHR: National Institute for Health Research
PPI: Patient and Public Involvement
RDS: Research Design Service
UK: United Kingdom
