# Supplementary material for: The mutual benefits of patient and public involvement in research: an example from a feasibility study (MoTaStim-Foot)
Source: Res Involv Engagem. 2021 Dec 4;7:87. doi: 10.1186/s40900-021-00330-w (PMC8645133; doi:10.1186/s40900-021-00330-w)
Supplement: Supplementary file 1 — Additional file 1. The GRIPP 2 checklist. [file 40900_2021_330_MOESM1_ESM.docx]

**Additional information 1: GRIPP2 short form**

From: [GRIPP2 reporting checklists: tools to improve reporting of patient and public involvement in research](https://researchinvolvement.biomedcentral.com/articles/10.1186/s40900-017-0062-2)

Referenced from Staniszewska et al (2017)^1^

| **Section and topic** | **Item** | **Reported on page No** |
| --- | --- | --- |
| 1: Aim | Report the aim of PPI in the study | 4–5 |
| 2: Methods | Provide a clear description of the methods used for PPI in the study | 5–9 |
| 3: Study results | Outcomes—Report the results of PPI in the study, including both positive and negative outcomes | 10–19 |
| 4: Discussion and conclusions | Outcomes—Comment on the extent to which PPI influenced the study overall. Describe positive and negative effects | 19-22 |
| 5: Reflections/critical perspective | Comment critically on the study, reflecting on the things that went well and those that did not, so others can learn from this experience | 10–22 |

1. Staniszewska S, Brett J, Simera I, et al. GRIPP2 reporting checklists: tools to improve reporting of patient and public involvement in research. *Research involvement and engagement*. 2017;3:13. doi:10.1186/s40900-017-0062-2
